# Impact of Bacterial Vaginosis on Perineal Tears during Delivery: A Prospective Cohort Study

**DOI:** 10.1371/journal.pone.0139334

**Published:** 2015-11-06

**Authors:** Vincent Letouzey, Sophie Bastide, Daniela Ulrich, Laurie Beccera, Mariella Lomma, Renaud de Tayrac, Jean Philippe Lavigne

**Affiliations:** 1 Department of Obstetrics and Gynaecology, Nîmes University Hospital, Nîmes, France; 2 Department of Biostatistics, Epidemiology, Public Health and Bio-informatics, Nîmes University Hospital, Nîmes, France; 3 EA2415 University of Montpellier 1, Montpellier, France; 4 Department of Microbiology, Nîmes University Hospital, Nîmes, France; 5 National Institute of Health and Medical Research, U1047, University of Montpellier, Nîmes, France; State University of Maringá/Universidade Estadual de Maringá, BRAZIL

## Abstract

**Objective:**

Long term effects of perineal tears pose a major worldwide health issue for women during delivery. Since bacterial vaginosis is related to major obstacles in obstetrics the aim of this study was to determine the relationship between bacterial vaginosis and the occurrence of perineal tears during vaginal delivery.

**Methods:**

Between June 2013 and December 2013 pregnant women delivering after 37 weeks were recruited at one University hospital / tertiary care referral center in the course of this single-center, prospective cohort study. Bacterial vaginosis was assessed according to Nugent score method. Logistic-regression model was used to estimate odds ratios, adjusted for other risk factors to test the relationship between bacterial vaginosis and the occurrence of 1st to 4th degree perineal tears in women undergoing vaginal delivery.

**Results:**

A total of 728 woman were included, 662 analyzed with a complete Nugent Score of the vaginal swab. The prevalence of 1st to 4th degree perineal tears was 35.8% (95% Confidence Interval (95%CI) = [32.2; 39.6]). The presence of BV was not significantly associated to the incidence of perineal tears neither in the univariate analysis (crude Odds Ratio = 1.43; 95%CI = [0.79; 2.60]; p = 0.235) nor in the multivariate analysis (adjusted Odds Ratio = 1.65; 95%CI = [0.81; 3.36]; p = 0.167). Instrumental delivery was the most important risk factor for perineal lacerations.

**Conclusions:**

There is no evidence that vaginosis is a risk factor for vaginal tears.

**Trial Registration:**

ClinicalTrials.gov N° NCT01822782

## Introduction

Long term effects of perineal tears pose a major worldwide health issue for women during delivery. Women who sustain high degree perineal tears during delivery have a significantly reduced quality of life due to dyspareunia or faecal incontinence [1,2]. Therefore, vaginal tears are seen as major morbidity of vaginal childbirth. Risk factors of tearing are macrosomia, shoulder dystocia, instrumental deliveries, and previous perineal tears [3–5]. Medical interventions, such as mediolateral episiotomy, or efforts to minimize macrosomia are often employed in order to minimize high degree perineal tears and their consequences. Bacterial vaginosis (BV) is a well-known risk factor for pre-term birth and whether it plays a role in other obstetrical morbidities is still to be investigated[6–8]. The composition of the vaginal ecosystem is not static but changes over time and in response to endogenous and exogenous influences [9]. Regardless of the predominant bacterial species in the vagina of a healthy premenopausal woman, lactic acid production is crucial to maintain a healthy vaginal ecosystem. Indeed, acidic pH prevents the overgrowth of potentially pathogenic microorganisms [10]. During pregnancy two main types of abnormal vaginal microbiom may occur: anaerobic vaginosis (or bacterial vaginosis, BV) and aerobic vaginitis (AV). BV is defined as an alteration in the vaginal microbiom resulting in a large decrease in or total loss of lactobacilli, accompanied by a massive increase in the concentration of anaerobic and facultative anaerobic bacteria [11]. BV microflora include *Gardnerella vaginalis*, *Mycoplasma hominis*, *Bacteroides* spp.[12]. Its typical feature is the absence of inflammation, which distinguishes it from other pathologies such as candidiasis, trichomoniasis and AV [13,14]. The current gold standard to diagnose BV consists in the evaluation of morphotypes through Gram stain analysis with a standardized scoring method, the Nugent Score [15]. To date no study has investigated the impact of the BV on the incidence of vaginal tears during delivery. Alteration of the vaginal microbiom may result in an alteration of the immune response of the vaginal mucosa. We therefore hypothesized that women affected by BV might be at a higher risk of perineal lacerations during vaginal delivery. The aim of the study was to investigate whether women with BV have a higher risk of occurrence of perineal trauma compared to women with a normal vaginal microbiom.

## Materials and Methods

### Study design

This is a prospective monocentric observational cohort study. Ethic approval was obtained from the relevant regional or institutional ethics committee responsible for human experimentation (“Interface Recherche Bioéthique” /Institutional Review Board, IRB N°13/03-01). All participants gave verbal informed consent which was approved by the local ethics committee. Verbal consent was documented in the clinical patient file. All patients also received a written document where they could withdraw their consent from the study. The study was registered in the Clinical trial registry (Clinicaltrials.gov N° NCT01822782).

### Settings and Subjects (Participants)

The study was carried out at Department of Obstetrics and Gynaecology of the local University Hospital, between June 2013 and December 2013. Eligible were all women aged between 15 and 45 years and with a gestational age ≥37 weeks undergoing vaginal delivery and for whom vaginal swab (VS) was analyzed at Department of Microbiology of the University Hospital.

### Outcomes

The main objective of the study was to evaluate a possible relationship between BV and the occurrence of 1st to 4th degree perineal tears. The rate of perineal tears was compared between the reference group (no BV) and the risk group (BV) to see whether BV increases the risk for perineal trauma. Since the BV mainly affects the mucosa and thus likely to have an effect on low grade perineal tears, a complementary objective was to analyses the relationship between BV and first degree perineal tears alone.

VS analysis is routinely performed in our patients according to international guidelines to evaluate the presence of Streptococcus agalactiae. All swabs were performed at the 35th week of gestation; symptomatic women that needed antibiotic treatment before, the vaginal analysis was performed, were not excluded. Nugent score was calculated as described before [15]. Briefly, Gram-stained smears are evaluated for their morphotypes (large Gram-positive rods, Lactobacillus spp; small Gram-variable rods, G. vaginalis; small Gram-negative rods, Bacteroides spp; curved Gram-variable rods, Mobiluncus spp; and Gram-positive cocci); each morphotype is quantitated from 1 to 4 with regard of morphotype per field (Table 1). To eliminate a bias of Gram lecture, a unique specialized microbiologist determined the Nugent Score. Depending on the result of the VS, women were divided into two groups; the test group (BV) with a Nugent Score greater ≥ 7 and the control group (no BV) with a normal vaginal microbiom (Nugent Score < 7). The obstetric staff was blinded to the Nugent score during delivery, only the *Streptococcus agalactiae* state was known.

**Table 1:** Nugent scoring system.

Score	*Lactobacillus* morphotype	*Gardnerella* and *Bacteroides* spp morphotypes	Curved Gram-variable rods
0	4+	0	0
1	3+	1+	1+ or 2+
2	2+	2+	3+or 4+
3	1+	3+	
4	0	4+	

Morphotypes are scored as the average number seen per oil immersion field (Note that less weight is given to curved Gram-variable rods). Total score = lactobacilli + *G*. *vaginalis* and Bacteroides + curved rods. 0 = No morphotypes present; 1 = <1 morphotype present; 2 = 1 to 4 morphotypes present; 3 = 5 to 30 morphotypes present; 4 = 30 or more morphotypes present.

### Data collection

Demographic and obstetric data were collected from hospital records at time of inclusion. After delivery, the perineum was inspected by a midwife and a medical doctor and the degree of the tear noted. The following obstetric parameters were also collected: time of first and second stage of delivery, mode of delivery, episiotomy, birthweight, and any event during delivery that could influence the risk of perineal tears.

### Sample size calculation

As no data or information on the prevalence of vaginal lesions depending on the nature of the vaginal microbiom was available, the sample size was determined to ensure a minimal statistical power of 80% to detect clinically relevant risk factors (relative risk ≥ 1.5). With a hypothesis of 35% prevalence of vaginal lesions and a frequency of BV equal to 10% (both estimated from registry data of the Nîmes University Hospital in 2011: overall prevalence of vaginal tears 38% and BV frequency to 10%), 660 patients were required (with a 2-sided type 1 error rate of 5%).

### Statistical analyses

Quantitative data are expressed as mean ± standard deviation (SD) or median with 25th and 75th percentiles and compared using t-test or Mann Whitney-U test, both according to the variable distribution. Qualitative variables are expressed as frequency with percentage and compared using chi-square test (or Fisher exact test if necessary); 95% Confidence Intervals (95% CI) were estimated using the binomial distribution exact method. A logistic regression model was used to find predictive factors of 1st to 4th degree perineal tears; first in a univariate analysis, in order to find potential confounders, and subsequently in a multivariate analysis, in order to adjust the analysis on the confounders identified. The results were presented using crude Odds Ratio (cOR) and adjusted Odds Ratio (aOR), with their 95% CI, for univariate and multivariate analysis, respectively. A complementary analysis to assess predictive factors of first degree perineal tears was performed with the same method. Sensitivity analysis was performed to assess the impact of missing values on the VS result with the assumption that missing VS were either all positive for BV or all negative for BV.

All analyses were performed using SAS version 9.4 (SAS Institute Inc., Cary, NC) using a 2-sided type 1 error rate of 5% as the threshold for statistical significance.

## Results

**Fig 1:**
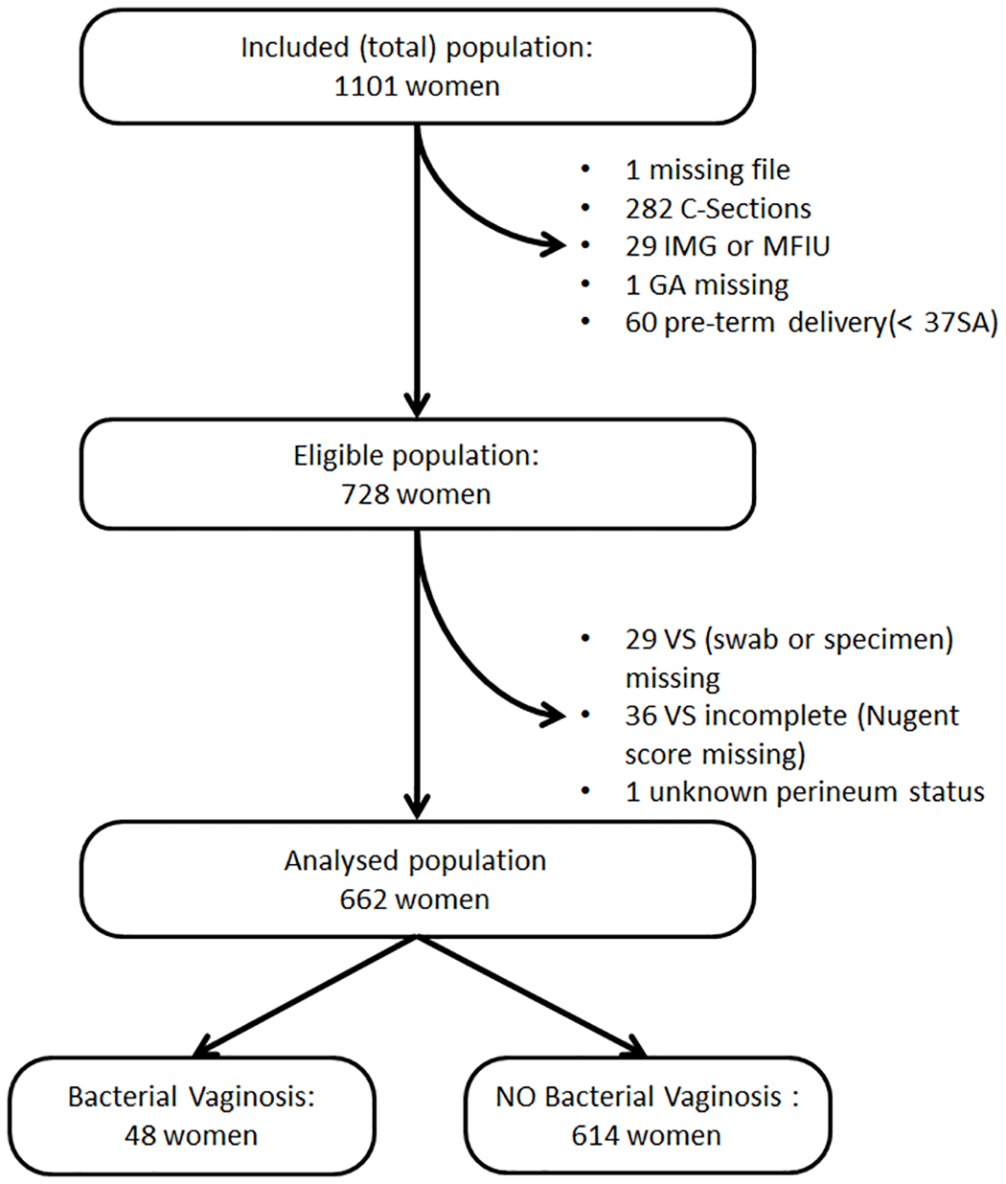
Flowchart describing the study population.

During the study period 728 women delivered vaginally after 37 weeks of gestation and were included in the study. Of these, 29 were excluded from statistical analysis for missing VS analysis, 36 for incomplete VS results, and 1 for unknown perineal status leaving 662 women for analysis (Fig 1). Patient characteristics of analyzed vs. non analyzed women did not reveal major or significant differences (Table 2). Mean age was 29.4 years old (± 5.6), 39.4% of women were primiparous, 19.9% smokers and 16.2% had gestational diabetes.

**Table 2:** Baseline data of non-analysed and analysed population within the study population (n = 728).

		Analysed		p-values
		No	Yes	
		(N = 66)	(N = 662)	
Age*	(n = 728)	30.1 ± 6.7	29.4 ± 5.6	0.397
BMI*	(n = 684)	27.8 ± 4.3	28.5 ± 4.9	0.321
Smoker^$^	(n = 669)	12 (23.5)	123 (19.9)	0.535
Parity^$^: primiparous	(n = 728)	20 (30.3)	261 (39.4)	0.147
Gestational Diabetes^$^	(n = 713)	4 (6.9)	106 (16.2)	0.061
Gestational Age^¤^	(n = 728)	39 [38–40]	40 [39–41]	0.015

BMI, Body Mass Index.

* mean ± standard deviation.

^$^ n (%).

^¤^ median [25^th^ percentile − 75^th^ percentile]

Forty eight out of the 662 (7.3%) analyzed women were positive for BV (95% CI = [5.4; 9.5]). Characteristics of the study population by groups are presented in Table 3. The noticeable clinical differences between groups were primiparity and the history of vaginal laceration (p = 0.002), the instrumental extraction delivery (p = 0.002), and the delays of the onset with active pushing but also the active pushing (p < 0.001). However, fetal presentation, head circumference or birth weights were not significantly different.

**Table 3:** Demographic and obstetric parameters of the analysed study population comparing women with and without 1^st^ to 4^th^ degree perineal tears.

		Perineal Tear	No Perineal Tear	p-value
		N = 237	N = 425	
Age*	(n = 662)	29.1 ± 5.3	29.6 ± 5.7	0.357
BMI*	(n = 631)	28.6 ± 4.8	28.5 ± 5.0	0.764
Smoker^$^	(n = 618)	41 (18.3)	82 (20.8)	0.453
History of perineal trauma^$^:	(n = 634)			
*Primipara*		109 (47.4)	152 (37.6)	0.002
*Multipara—Previous tear*		101 (43.9)	179 (18.1)	
*Multipara—No previous tear*		20 (8.7)	73 (18.1)	
Gestational diabetes^$^	(n = 647)	42 (17.9)	64 (15.2)	0.380
Previous use of antibiotics therapy^$^	(n = 655)	14 (5.9)	43 (10.3)	0.056
Presentation^$^:	(n = 657)			
*Occipito-anterior*		225 (95.7)	408 (96.7)	0.636
*Occipito-posterior*		9 (3.8)	11 (2.6)	
Instrumental Delivery^$^	(n = 662)	53 (22.4)	55 (12.9)	0.002
Episiotomy^$^	(n = 661)	13 (20.0)	92 (13.9)	< 0.001
Full dilation until active pushing (min.)¤	(n = 647)	75 [10–120]	45 [5–120]	< 0.001
Active pushing (min.)¤	(n = 652)	11 [6–21]	8 [4–18]	< 0.001
Streptococcal B colonization^$^	(n = 661)	29 (12.3)	73 (17.2)	0.096
Birth weight (g)*	(n = 653)	3319 ± 421	3315 ± 452	0.918
Head circumference (cm)*	(n = 639)	34.6 ± 1.3	34.5 ± 1.5	0.735

* mean ± standard deviation.

^$^ n (%).

^¤^ median [25th percentile − 75th percentile]

The prevalence of 1^st^ to 4^th^ degree perineal tears was 35.8% (n = 237/662; 95%CI = [32.2; 39.6]). More precisely, 92 patients (38.8%) had 1^st^ degree tears, 134 (56.5%) 2^nd^ degree tears, 11 (4.6%) 3^rd^ degree tears and none 4^th^ degree tears. The rates of the vaginal tears were 43.8% (n = 21/48; 9 1^st^ degree and 12 2^nd^ degree) and 35.2% (n = 216/614; 83 1^st^ degree, 122 2^nd^ degree and 11 3^rd^ degree) for women with and without BV, respectively. This corresponds to a relative risk of 1.24 (95%CI = [0.89; 1.74]). According to the univariate logistic regression analysis, the presence of BV was not significantly associated to the incidence of perineal tears (cOR = 1.43; 95%CI = [0.79; 2.60]; p = 0.235) (Table 4).

**Table 4:** 

	Univariate		Multivariate	
	cOR [95%CI]	p-values	aOR [95%CI]	p-values
Bacterial vaginosis	1.43 [0.79; 2.60]	0.235	1.65 [0.81; 3.36]	0.167
History of perineal trauma:				
*Primipara*	1		1	
*Multipara—Previous tear*	0.79 [0.56; 1.11]	0.175	0.63 [0.42; 0.96]	0.031
*Multipara—No previous tear*	0.38 [0.22; 0.66]	< 0.001	0.27 [0.15; 0.50]	< 0.001
Instrumental delivery	1.94 [1.28; 2.94]	0.002	6.09 [3.06; 12.12]	< 0.001
Episiotomy	0.07 [0.02; 0.18]	< 0.001	0.02 [0.01; 0.05]	< 0.001
Full dilation until active pushing (for 10 min.)	1.04 [1.01; 1.06]	0.006		
Active pushing (for 5 min.)	1.12 [1.04; 1.20]	0.004		

After adjustment on the identified confounding factors by the multivariate logistic regression analysis, the presence of BV remained no predictive factor for any degree of perineal tears (aOR = 1.65; 95%CI = [0.81; 3.36]; p = 0.167) (Table 4). The independent predictive factors of perineal tear were: the history of perineal trauma, operative vaginal delivery, episiotomy, and for the presence of *Streptococcus agalactiae*. Multiparity without a history of perineal tears, operative vaginal delivery and episiotomy resulted in the most important ones. Operative vaginal delivery was a high risk factor (aOR = 6.09; 95%CI = [3.06; 12.12]; p < 0.001) while multiparity without a history of perineal tears at the previous delivery were very protective (aOR = 0.27; 95%CI = [0.15; 0.50]; p < 0.001 and aOR = 0.02; 95%CI = [0.01; 0.05]; p < 0.001, respectively). All the results of the univariate and the multivariate logistic regression analysis are shown in Table 4.

Similarly to the primary outcome in the complementary analysis, first degree vaginal lacerations were not associated with the presence of BV (cOR = 1.48; 95%CI = [0.69; 3.16]; p = 0.316; aOR = 1.29; 95%CI = [0.57; 2.91]; p = 0.535 for univariate and multivariate analysis, respectively). The independent predictive factors for perineal tears were again the history of perineal trauma and the presence of *Streptococcus agalactiae*.

These results were confirmed by the sensitivity analysis: no significant association between BV and perineal tears was found, both with the assumption that all missing VS were positive for BV or negative (p = 0.822 and 0.208, respectively).

## Discussion

In a cohort of 728 pregnant women the prevalence of bacterial vaginosis (BV) was 7.8%; 237 women out of the 662 analyzed had perineal tears. According to the univariate and multivariate logistic regression analysis the presence of BV was not significantly associated to the incidence of all degrees of perineal tears (*p* = 0.235).

This study has several strengths. Firstly, to the best of our knowledge, this is the first study that aims to investigate the impact of the BV on perineal tears during vaginal delivery. Secondly, we included all pregnant women who referred to the Hospital between June and December 2013 and underwent vaginal delivery providing a large cohort of patients (662 women) required for the analysis. The timeframe of inclusions (6 months) was calculated based on the hospital birth registry and within this timeframe we successfully included the required number of patients. Importantly, of all women included none was lost to follow-up. Thirdly, we performed statistical analyses (both univariate and multivariate) of the relationship between BV and multiple degrees of tears (1^st^ to 4^th^). All previously known predictors for perineal tears (e.g. primiparity, instrumental delivery, delay of onset of active pushing and longer time of active pushing) were confirmed by adjusted analysis [3,4,16]. Fourthly, the current gold standard for detection and diagnosis of BV is Nugent score. We systematically use this method to analyses vaginal microbiom of all pregnant patients referring to the Hospital’s Obstetrics and Gynecology Department. This allowed us to include a significant number of patients in the study. Also, the three possible methodological types of bias were avoided: in terms of selection bias, over the six months period we prospectively included all eligible women; in terms of information bias, BV was defined objectively according to the Nugent score and the midwife that recorded perineal status data was blinded of the Nugent score of the patient; finally all confounders were adjusted by logistic model analysis. Finally, to address the problem of the missing VS and their potential impact on the results a sensitivity analysis was performed.

Our study has also some limitations. First, this is a single-center study that included all patients in a single Department of Gynecology and Obstetrics in which, as a tertiary maternity care, mainly low socio-economic women or complicated pregnancies are referred; therefore, the population represented may not be exhaustively representative. As recommended by international guidelines the use of episiotomies was restricted to few and specific cases to avoid severe maternal lacerations or facilitate complicated deliveries. In our analysis episiotomies were not considered as lacerations and thus, not included in the principal nor complementary endpoints. This may be seen as limitation of the study because if episiotomy would not have been performed, patient may still have undergone tearing but we cannot predict the severity. However, most recent studies agree on not considering episiotomies as vaginal lacerations[17]. Nevertheless, episiotomies were not excluded from analysis of independent risk factors for vaginal tears.

Vaginal tears diagnosis was made only after delivery in the delivery room and not confirmed by endoanal ultrasound in postnatal ward on the day after delivery.

Also, the BV prevalence observed in our cohort was actually lower than expected (7.3% vs 10%). This (prevalence) leads to a statistical power (67% in the a posteriori statistical power estimation) slightly lower than the 80% established to determine the required sample size. However, the observed effect didn’t reach the minimum effect size of clinically relevant factors we wanted to identify (RR = 1.2 vs RR = 1.5).

Alteration of the normal vaginal microbiom and in particular anaerobic BV has been linked to various pregnancy complications such as preterm delivery, premature rupture of the membranes (PROM), histologic chorioamnionitis, and infection of amniotic fluid [6,18,19]. Although a large number of different studies have explored the relationships between BV and pregnancy complications, none has so far focused attention on vaginal lacerations during delivery.

Importantly, BV is characterized by alteration of the vaginal microbiom and of the innate immune response of vaginal mucosa, which might be at risk of tearing during delivery. We hypothesized therefore, that women affected by BV might be at a higher risk of perineal lacerations during vaginal delivery. Furthermore, BV is a well-known risk factor for other pregnancy complications. Strikingly, our results clearly showed that the presence of vaginosis is not a risk factor for any stage of perineal tear. In particular tears of first degree, which affect the top layer of the skin and the vaginal mucosa were analyzed as complementary objective, and again no significant association with BV was found (Table 4). Recent studies have shown that molecular diagnostic tools are extremely powerful tool and they can provide new information about the composition of normal vaginal microbiota as well as abnormal colonization of the genital tract in both pregnant and non-pregnant women. In particular, Menard and colleagues have efficiently used quantitative PCR to characterize microbial dysbiosis in vaginas of pregnant women at high risk of preterm delivery[7]. With the same technique, they could also precisely identify ad quantify several microorganisms involved in vaginal microbiota anomalies showing that a high load of *Atopobium vaginae* alone is sufficient to identify women at risk of preterm delivery[8]. As molecular-based analysis techniques are more sensitive and powerful than Nugent scoring for identifying BV, we can hypothesize that they may as well uncover a link between BV (one or more particular bacterial species involved) and vaginal tears.

In a cohort of 728 women that delivered vaginally, the presence of BV had no impact on the outcome of the delivery in terms of vaginal tears (1^st^ to 4^th^ degree).
